# Using Individual Service Funds (ISFs) to Improve Access to Self‐Directed Support for Adults With Intellectual Disabilities: A Participatory Realist Review

**DOI:** 10.1111/jar.70148

**Published:** 2025-12-08

**Authors:** Elizabeth Croot, Alice Dunning, Andrew Booth, Clare Tarling

**Affiliations:** ^1^ Sheffield Centre for Health and Related Research, School of Medicine and Population Health Sheffield University Sheffield UK; ^2^ Clare Tarling Associates Dorchester Dorset UK

**Keywords:** individual service funds, intellectual disabilities, personal budgets, realist review, self‐directed support, social care

## Abstract

**Background:**

Individual service funds (ISFs) in England aim to provide self‐directed support without the challenge of procuring support and managing a budget. However, few local authorities offer ISFs and some do not offer more choice and control than a council‐managed budget.

**Methods:**

This participatory realist review followed RAMESES publication standards. We developed and refined theories using published and grey literature, expert stakeholders and personal narratives (written and video case studies).

**Results:**

We identified eight initial programme theories, forming a programme theory explaining how ISFs generate successful outcomes for adults with intellectual disabilities. These included: involvement in support planning; accessible budget information; flexible use of budgets; outcome‐focused support planning; ‘live’ support plans; non‐traditional support; positive risk management and trusting relationships.

**Conclusion:**

Our programme theory elucidates causal pathways for successful ISF outcomes, connecting mechanisms to contextual factors. This guides ISF development and implementation and helps adults with intellectual disabilities make informed decisions about ISFs.

## Highlights

- People with intellectual disabilities want to choose where they get support and who supports them. However, many people cannot or will not do this because buying their own support and managing their own budget is too complicated. This often means they have fewer choices about their support.
- ISFs were created to help more people have choice and control over their support. Even though The Care Act (2014) lists ISFs as one of the three main ways to get a personal budget, not many people are offered this option. Sometimes, local authorities say they are offering an ISF, but it is actually a council‐managed budget with a limited set of services. They're just calling it by a different name, which can be misleading.
- We worked with an advisory network, including adults with intellectual disabilities, and used research papers, reports, video case studies and interviews to develop explanations (called theories) about how ISFs should work for someone with intellectual disabilities.
- People who work in social care can use this to make sure their ISFs give someone more choice and control over their life. Adults with intellectual disabilities and their families can use this to check if the ISF they are being offered is likely to work well for them, helping them decide whether to accept the offer.

## 1 Introduction

The global movement towards individualised funding for adults with intellectual disabilities has gained significant momentum over the past 25 years, with numerous countries implementing systems that aim to shift control over funding for support arrangements from services to individuals (Carey et al. 2019; Dickinson 2017; Junne and Huber 2014; Kremer 2006). Individualised funding refers to systems where funding for support is allocated to the individual, rather than to services. This enables self‐directed support in contexts where individuals, their families or other advocates can exercise choice over how their funding is used to meet their needs.

This international trend reflects sustained advocacy by disabled people for greater control over their support arrangements and the recognition that traditional service‐led models often fail to deliver personalised outcomes (Disabled People's Organisations Forum 2021). Evidence from multiple countries demonstrates that self‐directed support improves outcomes, including greater involvement in support planning, increased control over support, enhanced access to services or activities, reduced support staff turnover and improved quality of life (DeCarlo et al. 2019; Harkes et al. 2014; Lakhani et al. 2016; Laragy and Fisher 2020).

The implementation of individualised funding takes various forms across different national contexts. In the UK, individual service funds (ISFs) were pioneered in Scotland in 1996 by Inclusion Glasgow, to provide flexible support for individuals with complex needs transitioning from institutional settings (Duffy 2014a). These early innovations influenced the development of Self‐Directed Support legislation in Scotland (Scottish Parliament 2013) and the Care Act 2014 (The Care Act 2014) in England, which formally recognised ISFs as a mechanism for delivering personalised support. Similarly, Australia's National Disability Insurance Scheme (NDIS) embodies comparable principles of individualised, person‐centred funding for people with disabilities (Australian Government: Department of Health 2025). European countries have developed parallel models: the Netherlands' Persoonsgebonden Budget (PGB) provides individuals with direct control over their care funding (Kremer 2006), whilst Germany's Persönliches Budget operates on similar principles, offering people with disabilities greater autonomy over their support arrangements (Federal Ministry of Labour and Social Affairs (BMAS) 2008).

In England, individualised funding is operationalised using personal budgets. Adults with intellectual disabilities who are eligible for state‐funded social care have a legal right for their needs to be assessed (Gosse et al. 2017), and a budget allocated for support to meet those needs (The Care Act 2014). This personal budget can be taken as a direct payment, where the individual, their family, or another intermediary, holds the budget and decides which support services will best meet their needs. Alternatively, all or part of the budget may be retained by the local authority and used to fund places in services procured under a block contract to meet community needs, or sometimes a mixture of the two. (Carr 2010; Department of Health and Social Care 2022; Dew et al. 2013). Direct payments offer greater self‐directed support opportunities and have demonstrated improved outcomes compared to council‐managed budgets (Glendinning et al. 2008; Hatton et al. 2006; Hatton and Waters 2011). However, most eligible adults with intellectual disabilities opt for council‐managed budgets (68,265) over direct payments (28,875), potentially limiting their access to the benefits of self‐directed support (NHS Digital 2022–2023).

This preference for council‐managed budgets may be for many reasons. Direct payments require individuals to navigate intricate care structures and advocate for their own rights and needs or rely on support from their social networks. Consequently, direct payments may be inaccessible to some individuals due to variations in the personal capabilities and resources necessary to negotiate complex, bureaucratic service systems (Gardiner and Iarocci 2018; Lorenc et al. 2013). Those who lack the requisite skills or resources to understand the intricacies of social care support may be less likely to opt for direct payments despite the greater opportunities for self‐directed support and the limited choice and control offered by council‐managed budgets (Hall 2009; Harkes et al. 2014; Needham 2013; Rabiee et al. 2009). Therefore, although direct payments offer opportunities for self‐directed support, they have the potential to exacerbate inequalities in care if they remain inaccessible to some people (Carey et al. 2019; Harkes et al. 2014; Malbon et al. 2019).

**FIGURE 1:**
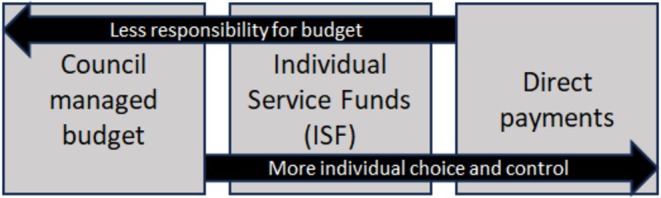
Relationship between direct payments, council‐managed budgets and ISFs.

ISFs have the potential to address this inequity by offering adults with intellectual disabilities the benefits of self‐directed support without the challenge of managing a budget (Figure 1).

### 1.1 What Is an Individual Service Fund (ISF)?

An ISF is an arrangement where a local authority contracts with a support provider to use an internal system of accounting that makes the budget transparent to the individual and their support circle, enabling them to decide how best to use the budget to meet their support needs in line with their preferences and priorities (Duffy 2014b). They are described in the Care Act guidance as one of the three main ways in which a personal budget can be used (Department of Health and Social Care 2022). However, 10 years on from the Care Act, they are still not widely available or understood despite the potential they offer adults with intellectual disabilities to benefit from individualised funding and self‐directed support (Fleming et al. 2021).

The reasons for this are multifaceted. ISFs are complex interventions (Moore et al. 2019), which are implemented within existing social care systems and involve multiple activities and processes, operating at multiple levels and depending on dynamic and evolving interactions and relationships, between different actors within the adult social care system (Hawe et al. 2009). Furthermore, adult social care settings are characterised by a combination of formal rules and established practices which function alongside more informal shared understandings about the values and expectations within that system (Carey et al. 2017). Interactions take place in different settings, each of which must function autonomously but is also subject to external constraints (Lakhani et al. 2016; Moore et al. 2019). As a result, there is a wide variation in the way that ISFs are understood and implemented across local authorities offering them, and this extends to the individual level, with adults with intellectual disabilities experiencing and using ISFs in distinct ways depending on their individual needs and circumstances.

Whilst flexibility is essential to the implementation of ISFs across different contexts, allowing them to be tailored to specific circumstances, the lack of consistency or fidelity to a specified plan makes them difficult to implement and evaluate (Hasson 2010; Lipsey and Cordray 2000; Morrison et al. 2009). Consequently, there is little research into their use, and questions remain about how ISFs are working and how they could be improved.

This review seeks to address this gap using a participatory realist approach to draw from diverse sources of evidence to develop programme theory. Programme theories are specific to an intervention, even though they may share much in common with other interventions (Davidoff et al. 2015). They are concrete working models rather than abstractions and their purpose is to describe the ‘theory of change’ (Weiss 1995), that is the rationale and assumptions about the causal pathways, expressed as context–mechanism–outcome configurations (CMOCs), by which the intervention generates both intended and unintended outcomes (Pawson and Tilley 1997). They provide a valuable tool for policy makers and practitioners looking to implement and evaluate ISFs across different settings by identifying elements that can be adapted whilst ensuring that mechanisms that are core to the intervention's success are understood and maintained (Fletcher et al. 2016; Hawe et al. 2004). Moreover, programme theories illuminate the contextual factors (Greenhalgh and Manzano 2022; Rojas‐Garcia et al. 2020) that are conducive to a successful ISF, thus enabling individuals and their advocates to assess the specific circumstances of an ISF offer, and to decide the extent to which these are likely to yield positive outcomes, and thereby informing decisions about whether to choose an ISF or alternative support arrangements.

ISFs have the potential to reduce inequalities in access to flexible support for adults with intellectual disabilities; however, it is important to explore the conditions under which people are more or less likely to benefit from them and why. This knowledge is essential for shaping policies that govern ISF development and delivery, and for informing decisions by individuals and their advocates about ISF uptake. Our review aimed to develop programme theory to help social care policymakers better understand and support the conditions necessary for ISFs to work effectively for adults with intellectual disabilities.

## 2 Methods

Realist reviews draw from multiple sources of evidence, including published literature, policy and guidance documents and expert stakeholders (Booth et al. 2019). They aim to explain how, why, for whom and in what circumstances a complex intervention, such as an ISF, works well (Pawson and Tilley 1997). Realist inquiry is theory‐driven and operates on the foundational principle that the delivery of intervention resources in a particular context generates adaptive changes in reasoning and behaviour, called mechanisms, which cannot usually be seen or measured but which result in observable outcomes (Dalkin et al. 2015; Jagosh 2019). Mechanisms are shaped by contingencies within the environment, which may enable or disable their causal action. It is this sensitivity to variation in circumstances that enables a nuanced exploration of the context in which mechanisms are activated, remain dormant or result in unanticipated outcomes (Greenhalgh and Manzano 2022). By focusing on contexts and mechanisms rather than specific procedures, this review aimed to produce transferable knowledge about how ISFs are hypothesised to work, which can be adapted to different settings (Moore et al. 2019). This is particularly valuable in the study of ISFs, given the considerable variation in the way these are configured across local authorities, and significant diversity among the individuals who use them (Rojas‐Garcia et al. 2020).

### 2.1 Design

We designed this review with an advisory network made up of adults with intellectual disabilities (*n* = 13), support workers (*n* = 4), family carers (*n* = 1), adult social care commissioners (*n* = 2), social workers (*n* = 2) and service providers (*n* = 2), who were recruited based on their experience of ISFs. The network operated as two groups that met every 2 months throughout the study. Adults with intellectual disabilities and two support workers from an existing self‐advocacy group had been involved in the development of the research idea and met face to face to discuss broader topics related to the delivery of ISFs. The remaining members were recruited from national self‐directed support and ISF networks and met online as a group to advise about the review process. Co‐developed terms of reference for the network included the use of Easy Read materials, simplified agendas with one to two items per meeting, regular breaks and plain English explanations of jargon terms or complex ideas. Members were paid for preparation time and additional support if needed.

We combined a desk‐based realist review, reported following RAMESES publication standards (Wong et al. 2016), with primary data from focus groups and individual interviews with key stakeholders. Stakeholders included adults with intellectual disabilities, family carers, support workers, support providers, social workers and adult social care commissioners. In view of the paucity of literature from the perspective of adults with intellectual disabilities who use ISFs, we also used online written and video personal narratives (case studies) as sources of evidence.

Realist approaches use iterative cycles of theory gleaning, refining and testing (Manzano 2016) and we carried these out in three cumulative phases consisting of: phase 1, a broad search for peer‐reviewed and grey literature, with input from an advisory network to develop initial programme theories (Pearson, Brand, Quinn, et al. 2015); phase 2, a focused search for evaluative evidence from literature and online written or video narratives to support, refute and expand our developing theories; and phase 3, qualitative research including individual interviews with key stakeholders and focus groups with adults with intellectual disabilities and family carers to further test and refine our theories.

### 2.2 Phase 1 Developing Initial Programme Theories

Phase 1 began with an exploratory scoping search of published and grey literature and policy documents using broad search terms, including individual service fund, personal budget, person‐centred planning and personalisation. Our aim was not to carry out a comprehensive search, but rather to identify potentially valuable insights into the putative mechanisms that contribute to the success of these approaches, as advanced in descriptions of how they are planned and expected to work.

We conducted a broad initial search across three databases: a social care (King's Fund), health (PubMed) and a general multidisciplinary database (Web of Science) to capture diverse potentially relevant papers. Specifically, we looked for papers that explicitly discussed theories or mechanisms related to ISFs or personal budgets to reflect contributions from disciplines or theoretical traditions and that might offer contrasting views or explanations. In this connection, it proved important to include policy documents, programme reports, or other non‐academic sources that might offer valuable insights into how ISFs are thought to work. Eighteen sources were identified initially. We extracted from these key sources against the realist concepts of context, mechanism and outcome in the form of if‐then‐leading to statements (Pearson, Brand, Quinn, et al. 2015). If‐then‐leading to statements are used in realist approaches to articulate initial programme theories. These statements expressed our initial ideas about causal pathways through which ISFs were thought to work: **if** a particular context (C) is present, **then** it generates specific mechanisms (M) **leading to** particular outcomes (O). See Table 1. Where individual sources only yielded partial accounts, we reviewed and discussed interrelationships and overlaps across multiple sources to link these within an initial set of statements. Successful outcomes of quality social care support were mapped against six objectives for people who draw on care and support, unpaid carers and professionals who provide care and support, included in the adult social care outcomes framework (ASCOF 2023–2024). These were: quality of life; independence; empowerment, information and advice; safety; social connections; and continuity and quality of care. Through this process of theory gleaning, sources that did not contribute anything unique to the statements were removed, leaving 14 sources, including peer‐reviewed research (Beadle‐Brown et al. 2017; Carey et al. 2019; Carson and Hoolahan 2015; Conder and Mirfin‐Veitch 2020; Fleming et al. 2019; Leverton et al. 2022; Malbon et al. 2019; Manthorpe et al. 2020; O'Brien and Randjelovic 2024; Ratti et al. 2016; Robinson et al. 2022), guidance documents (Duffy 2014a; Self‐Directed Futures 2016) and reports (Duffy 2008).

**TABLE 1:** Example of an if‐then‐leading to statement generated during phase 1.

**IF** support providers know their clients' preferences and priorities, understand the outcomes that they are working towards and have a good knowledge of the resources available in the wider community, **THEN** they will be able to identify and offer relevant support options, **LEADING TO** greater opportunity for the individual and their families to choose support options and activities that are meaningful to the individual (Carson and Hoolahan 2015; Duffy 2008, 2014a; Ratti et al. 2016; Robinson et al. 2022).

Our advisory network contributed to discussions about the emerging if‐then‐leading to statements, adding further context for the reviewers and supporting the process of completing partial statements. Advisory network meetings were audio‐recorded and extensive notes were made and used to refine, expand and add to the if‐then‐leading to statements using a process involving deduction, induction, abduction and retroduction (McEwan et al. 2023; Mukumbang et al. 2021).

At the end of phase 1, if‐then‐leading to statements were discussed, compared and grouped into four conceptual buckets (Bender et al. 2021), according to the ideas within them; these were: individualised budgets, person‐centred support planning, asset‐focused support and outcome‐focused support.

By comparing statements in each conceptual bucket, we were able to further refine, expand or complete statements to build more comprehensive initial theory statements identifying the contexts and mechanisms by which each component was thought to achieve successful outcomes; see Table 2. In this way we developed initial theories about how ISFs ‘should’ work.

**TABLE 2:** Example initial theory—asset‐focused support.

Families and friends can be a source of good ideas, local connections and creative support solutions. Context—the adult with intellectual disabilities has family and friends who are involved in their lives and the support provider has a good relationship with the individual and their family and friends. Mechanism—they work together to identify support opportunities and resources within the adult with intellectual disabilities' network, and the local community. Outcomes: Greater involvement of the individual and their family and friends in support planning (Links to ASCOF: Independence; building connections with family, friends and their community; empowerment; control) Improved communication between providers and family and friends (Links to ASCOF: Building connections with family, friends and their community) Reduced anxiety about the support plan (Links to ASCOF: Safe support which meets needs; Empowerment) Opportunity to develop creative support plans that make use of existing community resources to meet the needs of the individual whilst promoting greater involvement of people with intellectual disabilities in their local communities (Links to ASCOF: building connections with family, friends and their community; independence; quality of life) Improved satisfaction with support (Links to ASCOF: Quality of life)

### 2.3 Phase 2: Focused Evidence Retrieval and Synthesis

In phase 2, we carried out focused systematic searches for evidence that linked contexts and/or mechanisms with outcomes, to enable us to test and further develop the initial theories generated in phase 1. We searched published and grey literature for pilot or ISF evaluations. We supplemented this with a separate search for evidence about ISFs from the perspective of adults with intellectual disabilities. This search used domain searching (e.g., site:org.uk) on Google to scrutinise the websites of organisations offering or supporting ISFs and elicited pertinent evidence in the form of multimedia narratives, including videos and written accounts of experiences of ISFs.

We searched four search platforms, Google Scholar, Elicit (https://elicit.com/), LitSense (https://www.ncbi.nlm.nih.gov/research/litsense/) and Consensus (https://consensus.app/home/blog/welcome‐to‐consensus/), in August 2023. Search methods drew on guidance for the realist search (Booth 2019; Booth et al. 2019, 2018; Dada et al. 2023). Theory testing for realist reviews requires identification of data at a high level of granularity so that it specifically addressescontext ‐mechanism‐outcome components, (CMOCs) (Graham and McAleer 2018). For example, we searched for ‘asset focused support’ rather than the broad topic area ‘Individual Service Funds’. This granularity cannot be sustained by conventional title and abstract‐based searching (Coleman et al. 2020), for example, ‘asset focused support’ would register zero results in PubMed, and so our search required access to full‐text via Google Scholar and through citation‐in‐context systems such as the three named here (Allot et al. 2019).

Elicit, LitSense and Consensus represent a new generation of AI‐powered research platforms that significantly enhance realist review methodology beyond conventional bibliographic databases. These platforms enable semantic searching that can identify relevant content regardless of specific terminology used, allowing us to identify information about context‐mechanism‐outcome components directly from full texts. This is particularly valuable for realist reviews because the methodology requires developing programme theories that explain ‘what works, for whom, in what circumstances and why’ through CMOCs (Graham and McAleer 2018).

For complex interventions like ISFs, where mechanisms may operate differently across various contexts and populations, these platforms' ability to extract granular information about contextual factors, mechanism descriptions and outcome variations from large volumes of literature significantly enhances the efficiency and comprehensiveness of evidence synthesis. The semantic search capabilities also help identify relevant evidence that might be missed through traditional keyword approaches, which is particularly important when dealing with interventions that may be described using different terminology across different disciplines or systems.

We searched using keywords derived from each programme theory, for example, ‘asset focused support’ AND words relating to individualised or personalised budgets or funds such that each combination linked the focal phenomenon (individual service budgets) to programme theory keywords criss‐crossing the literature with multiple topic areas. Whilst our focus was on UK‐based literature, where few or no items of literature were found, the search was extended to any relevant care system, for example, Australia.

#### 2.3.1 Selection and Appraisal of Sources

Database search results were transferred into Excel and independently screened by two reviewers (AD, CW). A subset (20%) of the sources was screened by multiple reviewers (AD, CW, AB, LC) to check the consistency of screening decisions. Following realist synthesis principles, sources, including written and video case studies, were included if they contained rich and relevant insights (Dada et al. 2023) to link ISFs to outcomes of support. All reviewers met to deliberate on the inclusion and the quality of the identified sources, whilst also identifying gaps in the evidence that necessitated further searching.

#### 2.3.2 Data Extraction and Synthesis

A data extraction form was developed, piloted and refined through discussion within the team. This included: source context (study, ISF pilot, case study), participants and evidence relating to theories and outcomes. Data extraction was carried out by three designated reviewers (AD, CW, LC), with a subset for each subjected to verification by a second reviewer. Reviewers used a deductive approach to extract data to either support, refute or refine theories, alongside an approach integrating induction and retroduction to uncover any new data. This process was iterative and non‐linear and the reviewers met with the advisory network to discuss the process and decisions made to refine and develop initial theory statements; see Table 3.

**TABLE 3:** Example of an expanded theory statement at the end of phase 2.

If support providers: Understand and explain to the individual and their family and friends what an ISF is and how it can be used Know the individual's preferences and priorities Understand the outcomes that they are working towards Listen and share ideas about support with the individual and their family and friends Have a good working knowledge of the community Support the individual to try new things Then they will be able to: Identify and offer a genuine choice of support options that will address the outcomes that are important to the individual in ways that are appealing to them, and to support the individual to make choices and try new things. Leading to: Opportunity to exercise genuine choice New experiences and opportunities for the adult with intellectual disabilities Support that is embedded in the local communities and therefore helps them build community connections Support that works towards outcomes that are important to the individual and their families and friends

### 2.4 Phase 3: Testing and Refining of Programme Theory

In phase 3, we tested and further refined our programme theory using individual interviews and focus groups with key stakeholders. With informed consent, we carried out individual interviews with eight stakeholders with experience of delivering ISFs from our advisory network, and held four online focus groups, two with adults with intellectual disabilities (*n* = 15) and two with family carers of an adult with intellectual disabilities who had been offered or used an ISF (*n* = 6).

We received ethical approval from the University of Sheffield, School of Health and Related Research ethics committee (20/07/23; REF: 054823) to conduct these interviews and focus groups.

#### 2.4.1 Data Collection Procedures and Accessibility Adjustments

The interview and focus group topic guides were based on our developing theories and aimed to elicit stakeholders' experiences and provide a deeper understanding of the way ISFs are thought to work to produce positive outcomes. Topic guides were developed and piloted with our advisory network, and questions were tailored to the participants' experience; for example, questions for family carers focused on their experience of support for their adult offspring, whereas questions for those delivering ISFs focused on delivery.

Individual interviews and focus groups with carers were carried out online using Google Meet (the University's preferred platform).

Focus groups with adults with intellectual disabilities were carried out with members of a pre‐existing self‐advocacy group selected for the diversity of members' support needs and ethnic backgrounds. This group used Zoom video conferencing for weekly meetings, and was familiar with the platform so these focus groups used Zoom. Some members joined independently whilst others had support to enable them to participate.

Focus groups with adults with intellectual disabilities used accessible language to help participants link the topics to their own experiences so they could contribute in a meaningful way. We developed and piloted our data collection method with our advisory network, who advised about appropriate language, for example, ‘changing your mind’ rather than ‘flexibility’ when exploring what contributes to good support planning. We developed a visual method, using line drawings of a person, to improve accessibility and focus conversations about support. This allowed participants to distance discussion from their personal experiences if they wanted to. The group named and described the person before discussing different support scenarios chosen to align with our emerging ideas. This method worked well, and the groups engaged with enthusiasm.

#### 2.4.2 Data Analysis

All interviews and focus groups were recorded, and extensive notes were made. The data were analysed deductively using the initial programme theory as a framework. Data relating to context, mechanisms, or outcomes of ISFs for each theory area were summarised in a table with a separate column for notes regarding theory refinement; for example, the inclusion of a new outcome. Table 4 provides examples of supporting evidence from stakeholder interviews and focus groups for programme theories.

**TABLE 4:** Examples of supporting evidence from stakeholder interviews and focus groups for programme theories.

*‘It's about local people, a small team working around somebody in a network. That's embedded in the community where you're drawing on natural networks of support and you're doing everything that you can to kind of look at what the person can do, what friends and family, what the community can do, assistive tech with paid staff being the very last resort.’* [Provider 2—Asset‐focused support planning]
*‘I think it depends on the provider and the buy‐in. So we've got one particular provider who really does understand the delivery of ISFs, what they do is they get groups of people together who enjoy being supported to do activities, for example, going to the gym or swimming or, it might be community meal and they'll tap into other individual, bespoke elements.’* [Commissioner‐ flexible use of budgets/joint purchasing]
One person described their own bad experiences in the past in a setting where people were not nice, and so they felt ‘*isolated’* and *‘helpless’*. They made the decision not to continue going to this setting with the support of their parents. The group spoke about the person in our picture, and they named him Rodger. Rodger can choose what activities he wants to do, like swimming and art therapy, and so he is the *‘happiest person’*. Being able to make decisions about activities and stop going to places he did not like has turned his life around. He used to feel *‘depressed’*, not *‘happy’*, but changing things means people *‘feel better in themselves’*. [Focus group, adults with intellectual disabilities, notes—Support that is reviewed regularly and changed easily]
Carers said that ISFs that offer the flexibility to make changes without having to go back to the local authority work well. Flexibility is important because being able to make quick changes in response to a change in need allows for earlier intervention, which can prevent or delay needs from escalating. [Focus group, carers, notes—Support that is reviewed regularly and changed easily]

Following the collation of the evidence from stakeholders, the review team held several meetings to discuss the findings from the interviews and focus groups alongside the emerging theories. We held regular meetings with the advisory network which contributed to emerging interpretations and guided the development of theories. Using an in‐depth process that included deduction, induction, abduction, retroduction and comparison, we synthesised data from the literature with that from the interviews and focus groups to expand, refine and develop existing theories, resulting in a set of initial programme theories.

## 3 Findings

**FIGURE 2:**
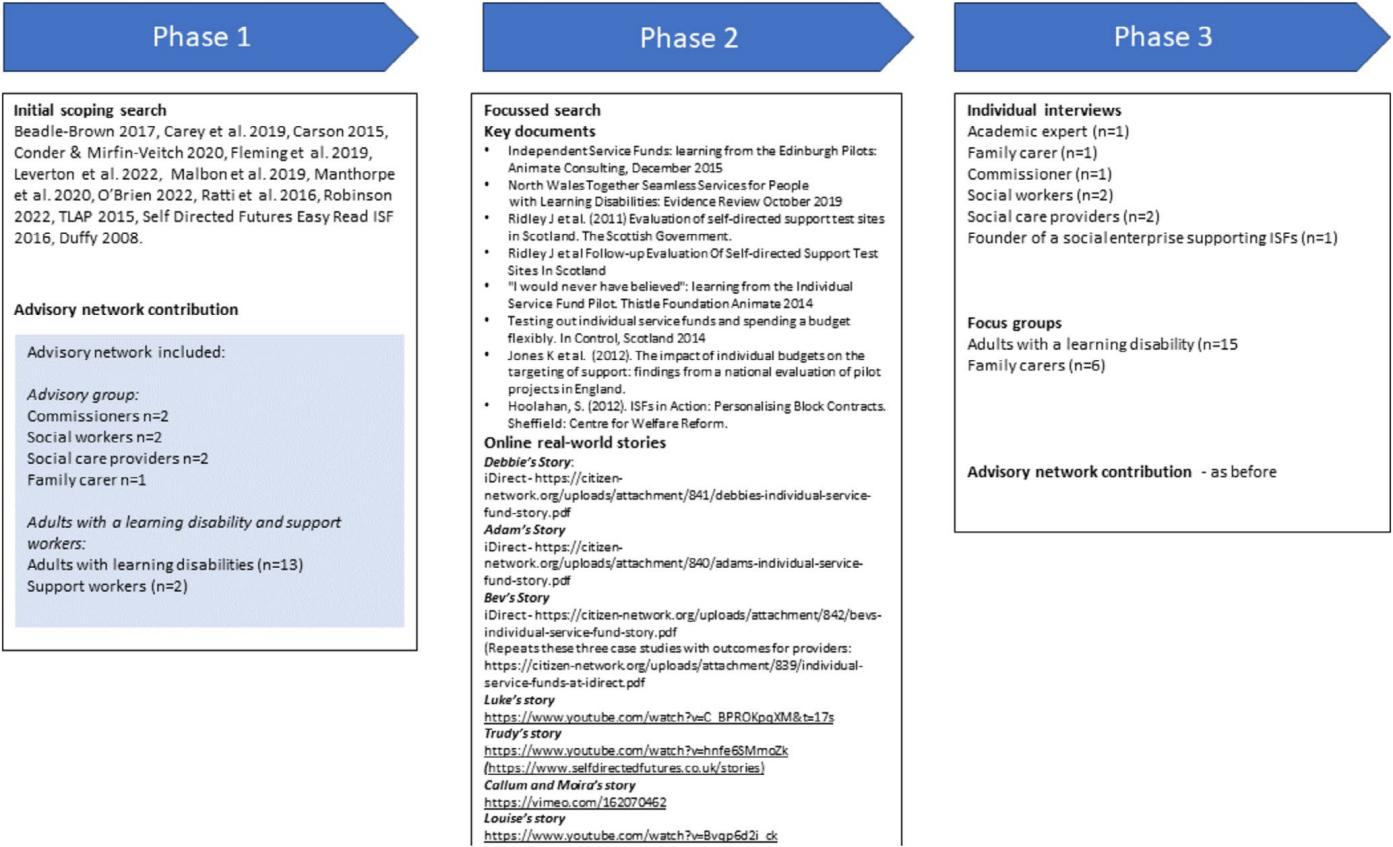
Processes and literature searches used to develop the programme theory.

Our final set of theories was drawn from 14 papers from our initial search and eight papers from our focused search for ISF pilots or evaluations, seven online sources reporting real‐world stories, eight individual interviews and two focus groups each with adults with intellectual disabilities and family carers, along with multiple inputs from our advisory network of adults with intellectual disabilities, support workers, social care commissioners, social workers, social care providers and family carers; see Figure 2. From this point, we use the term ‘advocates’ to include family carers as well as any others who make up an individual's support network.

### 3.1 The ISF Programme Theory

We identified eight initial programme theories that together make up the overarching programme theory; Table 5 summarises the sources that contributed to each of these theories.

**TABLE 5:** Overview of the context‐mechanism‐outcome configurations that make up the programme theory with supporting evidence.

Theory area	Theory	Supporting evidence
Involvement in support planning	1.1 The ISF provider involves the individual or their advocates in developing the support plan *Context*: The ISF provider makes sure the individual or their advocates are actively involved in deciding the support that is provided within the available budget to achieve the individual's outcomes *Mechanism offer*: The individual or their advocates have the opportunity to shape the support plan around the individual and incorporate their preferences within the constraints of the budget Mechanism response: The individual or their advocates feel confident the support plan is designed around the individual Outcome: The support plan is tailored to the individual's preferences and priorities The individual or their advocates are more satisfied with the support arrangements Trust is built between the provider and the individual or their advocates The individual or their advocates feel less anxious about the support arrangements 1.2 A positive experience of support planning builds trust and experience *Context*: The individual or their advocates have positive experiences of working closely with the provider because they see that the provider is listening to them and that the support plan reflects their preferences and priorities. *Mechanism offer*: The individual or their advocates have the opportunity to make suggestions about their support plan and they see that they are heard and their suggestions are implemented in the support plan. *Mechanism response*: The individual or their advocates realise that they have agency and opportunity to influence the support they receive so they offer their opinions. *Outcome*: The individual or their advocates gain experience in contributing to support planning and decision‐making The relationship between the provider and the individual or their advocates is strengthened	Focused search: Animate Consulting 2015; North Wales Together 2019, Ridley et al. 2012; Thistle Foundation Animate 2014 Online real‐world stories: Adam's story: https://citizen‐network.org/uploads/attachment/840/adams‐individual‐service‐fund‐story.pdf Debbie's story: https://citizen‐network.org/uploads/attachment/841/debbies‐individual‐service‐fund‐story.pdf Bev's story: https://citizen‐network.org/uploads/attachment/842/bevs‐individual‐service‐fund‐story.pdf Luke's story: https://www.youtube.com/watch?v=C_BPROKpqXM&t=17s Trudy's story: https://www.youtube.com/watch?v=hnfe6SMmoZk Callum and Moira's story: https://vimeo.com/162070462 Advisory network contribution: Advisory Group meetings (May. 23; June, 23; September, 23; November, 23; January, 24); Adults with intellectual disabilities/support worker workshops (July, 23; September, 23; October, 23); Carers' workshops (December, 23 a,b) Stakeholder interviews: Social enterprise founder, academic expert, provider, commissioner, provider, social worker, family carer Stakeholders example quote: It's about them being able to have a voice in what they want, or if they have really good representatives that really understand their wants and wishes, then that's when everything starts falling into place. Because if somebody's coming to me and going, I've got an idea, I want to do this, I need this support because if I don't have that, I can't do this. Then for me as a provider, I'm like this is brilliant. Great, this is how we can do that. (care provider manager participant 4, part 1 stakeholder interviews)
2 Transparent use of budget – information to decide support and to make changes if needed	2.1 Having accurate information about the budget in an accessible format *Context*: Adults with intellectual disabilities or their advocates are given accurate, clear and timely information about their budget and how it is being used, in a format that they can understand. *Mechanism offer*: The individual or their advocates have the information they need to weigh the different support options against the budget implications and to make a decision about how they would like to use the budget. *Mechanism response*: The individual or their advocates make decisions because they are confident they understand the financial implications of the choices they face about their support arrangements. *Outcomes*: The individual or their advocates feel able to make good decisions about the support package The individual or their advocates feel in control of the budget and the support arrangements Feeling in control and able to make good decisions leads to feelings of empowerment Receiving clear and accurate information from the provider reassures individuals or their advocates they are not being taken advantage of, this builds trust between the provider and the individual or their advocates Receiving information about the budget in an accessible format and being supported to use this information in decision‐making helps to develop the skills needed to manage a budget	Focused search: Animate consulting 2015; North Wales together 2019; Ridley et al. 2011 Online real‐world stories: Adam‘s story: https://citizen‐network.org/uploads/attachment/840/adams‐individual‐service‐fund‐story.pdf Debbie‘s story: https://citizen‐network.org/uploads/attachment/841/debbies‐individual‐service‐fund‐story.pdf Bev‘s story: https://citizen‐network.org/uploads/attachment/842/bevs‐individual‐service‐fund‐story.pdf Trudy‘s story: https://www.youtube.com/watch?v=hnfe6SMmoZk Callum and Moira‘s story: https://vimeo.com/162070462 Advisory network contribution: Advisory Group meetings (June, 23; July 2023; September, 23; November, 23); Adults with intellectual disabilities/support worker workshop (July, 23); Carers workshops (December, 23 a,b) Stakeholder interviews: Provider, family carer, social worker Stakeholders example quote: The person could know exactly how much was spent and how much was left…, you want to make these people more independent and you have no transparency for money, but are you crazy? This is ridiculous… So this is the point, transparency, there is not an obligation and this is really something that should be corrected. (family carer participant 2 stakeholder interviews)
	2.3 Support to make decisions about how to use the budget *Context*: The support provider knows the individual's abilities and their capacity to contribute to decision‐making. The provider has the time and the skill to explain the different support options and the financial and other consequences of each option, and to provide appropriate support to enable the individual or their advocates to make and express a choice. *Mechanism offer*: The individual or their advocates have the information they need in a format they can understand, and support to make or contribute to decisions in whatever way they can about how they use the available budget to meet their eligible support needs. *Mechanism response*: The individual or their advocates understand the choice of support options and the financial consequences of each choice, applied to their own situation and they are able to express their choice and thereby contribute to the decisions that are made in appropriate ways according to their capacity. *Outcome*: The individual or their advocates gain experience of making choices about support and the use of a budget. The support plan is based on the preference and priorities of the individual The support provider learns more about the individual's preferences and priorities when making a choice The advocates feel more confident that the support plan reflects the individual's interests and preferences The individual and their advocates have a positive experience of being supported and listened to because they see their choices reflected in the support plan.	We've also conditioned people to receive care in the same way for quite a long time, for most their lives. So then we're asking people to make decisions about how to use their budget differently. And they're not really even used to being able to make decisions because for so long they've been given 10 h a week or 20 and they go again to the day service and so a lot of that work, I think is then helping people understand what decisions are. (Social enterprise founder participant 1 stakeholder interviews)
3 Ability to use the budget flexibly	3.1 Local authority contract ‘allows’ providers to use the budget flexibly *Context*: the local authority contract allows the support provider to use the budget flexibility in the way they address the eligible support needs. *Mechanism offer*: the provider is permitted within the terms of their contract, to work with the budgets they hold to devise ways to offer innovative and varied activities and experiences to individuals, within their budget by saving, sharing or pooling budgets *Mechanism response*: the provider continuously looks for different and interesting ways to deliver/offer support and meet individual outcomes achieve. *Outcome*: the individual is offered more interesting and varied support options 3.2 The support provider actively looks for flexible ways to use the budget to enable a wider variety of different support options *Context*: The support provider is willing and able to be flexible, knows they have scope within their contract to use individuals‘ budgets flexibly, monitors the individual‘s progress, preferences and support needs and is actively looking for opportunities to offer more interesting and varied support options to address the needs of the individual *Mechanism offer*: the budget is managed in a way that enables a wider range of support options, activities or experiences to choose from, with appropriate support to try something new and a clear explanation of how this will be delivered using the budget, giving the individual or their advocates the opportunity to decide whether to make changes to their support plan. *Mechanism response*: The individual and their advocates feel confident they can use their existing budget to try something different in the way their support is delivered/provided/activities/experiences.	Focused search: Animate Consulting 2015; North Wales Together 2019, Ridley et al. 2011; Thistle Foundation Animate 2014 Online real‐world stories: Adam‘s story: https://citizen‐network.org/uploads/attachment/840/adams‐individual‐service‐fund‐story.pdf Debbie‘s story: https://citizen‐network.org/uploads/attachment/841/debbies‐individual‐service‐fund‐story.pdf Bev‘s story: https://citizen‐network.org/uploads/attachment/842/bevs‐individual‐service‐fund‐story.pdf Luke‘s story: https://www.youtube.com/watch?v=C_BPROKpqXM&t=17s Trudy‘s story: https://www.youtube.com/watch?v=hnfe6SMmoZk Callum and Moira‘s story: https://vimeo.com/162070462 Advisory network contribution: Advisory Group meetings (May. 23; June, 23; September, 23; January, 24); Adults with intellectual disabilities/support worker workshop (September, 23); carers workshop (December, 23b) Stakeholder interviews: Social enterprise founder, academic expert, provider, commissioner, social worker, family carer Stakeholders example quote: So it might be like, this afternoon, I might want to go to the pictures. I don‘t want to go to the pictures every single Wednesday afternoon, so it‘s about that ability to chop and change and being able to work as an individual with your ISF provider and their ability to be able to change. So having that autonomy, really and not to have to keep going back to your commissioner (commissioning manager, participant 6 stakeholder interviews)
	*Outcomes*: Opportunity to exercise choice across a wider range of options More interesting and varied support plans Opportunity to try new experiences and activities Build skills and experiences Greater opportunity to be involved in community activities More opportunities to meet new people Support plans more closely match the individual's skills and preferences Increased satisfaction that support is tailored to the individual 3.3 Support to make decisions about trying new experiences and activities *Context*: the individual and their advocates trust the provider and the provider actively seeks to support the individual to try new experiences and activities and offers encouragement and support along with reassurance that they can change their minds or try something else if they choose. *Mechanism offer*: the individual will have support to choose new activities allowing them to try new things and reassurance that they can change their mind or try something else if the new arrangement does not work well *Mechanism response*: the individual feels confident to voice their preferences and to try new things *Outcome*: The individual or their advocates have more opportunities to exercise genuine choice rather than selecting something because it is familiar The support plan is more varied and interesting The individual experiences new activities The individual and their advocates feel confident they have the opportunity to try new experiences and activities with appropriate support in place to do so and reassurance they can change again if things do not work well The staff delivering the support plan have a more varied and interesting job	Cafes and charity shops and different opportunities within the community. They may well shut down. We've had closures and where individuals have been doing voluntary work there, but can't anymore. and they've got so familiar with it and safe. They then become reluctant to try again and it's just about building back up their confidence about going No, you did this really well, you know how to do this, how to use a coffee machine. Or this is how you clear plates and then you wash up. It's just a different place. It's a different machine. (Care provider manager participant 4, part 2 stakeholder interviews)
4 Support planning based on outcomes not time and task	4.1 Building a support plan that is focused on the outcomes that are important to the individual or their advocates *Context*: the provider has a good understanding of the overarching outcomes and any proximal outcomes the individual is working towards, and their relative importance to the individual and their advocates. The provider also has good knowledge of the individual's support needs and the family and community resources available to the individual. The provider is motivated to continuously look for different activities and creative ways to work towards outcomes and they have the trust of the local authority to assess the risks and benefits of each activity on an individual basis. Mechanism Offer: The individual and their advocates are offered activities and support options that are tailored to their abilities and preferences and that will help them progress towards the outcomes that are important to them, using resources within their community. *Mechanism response*: The individual or their advocates choose from a range of relevant and feasible activities, experiences and support options that offer them the opportunity to progress towards the objectives that are important to them. *Outcomes*: The individual or their advocates have a genuine choice of relevant and feasible experiences, activities and support options The individual has the opportunity to try new activities and support options that allow them to be part of their local community The individual experiences a more interested, varied and purposeful support plan The individual makes progress towards the outcomes that are important to them, depending on the outcomes this might lead to increased independence, self‐ confidence, self‐determination and autonomy This might reduce the need for future support and help to prevent future needs The individual and their advocates feel more satisfied with the support they receive	Focused search: Animate Consulting 2015; North Wales Together 2019, Ridley et al. 2011, 2012; Thistle Foundation Animate 2014 Online real‐world stories: Adam's story: https://citizen‐network.org/uploads/attachment/840/adams‐individual‐service‐fund‐story.pdf Debbie's story: https://citizen‐network.org/uploads/attachment/841/debbies‐individual‐service‐fund‐story.pdf Bev's story: https://citizen‐network.org/uploads/attachment/842/bevs‐individual‐service‐fund‐story.pdf Luke's story: https://www.youtube.com/watch?v=C_BPROKpqXM&t=17s Trudy's story: https://www.youtube.com/watch?v=hnfe6SMmoZk Callum and Moira's story: https://vimeo.com/162070462 Advisory network contribution: Adults with intellectual disabilities/support worker workshop (July, 23) Stakeholder interviews: Social enterprise founder, provider, commissioner, social worker, family carer Stakeholders example quote Look at it more, broadly, what do you want to achieve in the next year? What you want to achieve in the next five years? What would make your life better? What are you really excited about for the future. So some of those such strengths focused and progressive thinking of where we want to get, not just the immediate problem or risk or not just being risk focused but actually focused on positive outcome and what would make life good for you? So, I think the ISF in turn will deliver better outcomes if you've thought like that (Social worker participant 7 stakeholder interviews)
5 Support plans as live documents that are reviewed regularly and changed easily	5.1 The support provider regularly reassesses the support plan and makes changes in response to changing needs *Context*: The support provider regularly reassesses progress towards outcomes, using both formal and informal methods, so they have a good understanding of the individual's abilities and preferences and can quickly offer adjustments to the support plan to add interest and opportunities for further progress. The contract between the local authority and the support provider gives the provider the authority to make changes to the support plan without the need for a formal review involving the local authority. *Mechanism offer*: The individual and their advocates are engaged in support planning that is proactive in response to changes in their abilities and support needs Mechanism Response: The individual or their advocates have the opportunity to make changes to the support plan in a timely way without the need to wait for a lengthy or anxiety provoking formal assessment *Outcomes*: The individual has a support plan that responds quickly to a change in circumstances or need and has the potential to prevent future needs escalating The individual and their advocates have the reassurance that the support plan can be changed if something does not work well	Focused search: Animate Consulting 2015; Thistle Foundation Animate 2014 Online real‐world stories: Adam's Story: https://citizen‐network.org/uploads/attachment/840/adams‐individual‐service‐fund‐story.pdf Debbie's Story: https://citizen‐network.org/uploads/attachment/841/debbies‐individual‐service‐fund‐story.pdf Bev's Story: https://citizen‐network.org/uploads/attachment/842/bevs‐individual‐service‐fund‐story.pdf Advisory network contribution: Advisory Group meetings (May. 23; June, 23; July, 23) Stakeholder interviews: Provider, commissioner, social worker, family carer Stakeholders example quote: And this, I want to point out, can be a very good positive tool because ISF is flexible because ISF is quick. I mean you don't have to go through different procedures with local authority or to call in social services and say, [son's name]'s needs changed, and now, we need more hours, we need a different workshop, we need a different support worker, he doesn't like the one he has. So this is for part that I suddenly discovered flexibility and quick changes (Family carer participant 2 stakeholder interviews)
6 Support plans make use of support networks and community resources	6.1 The support provider offers use of community assets in the support plan *Context*: The support provider has a good understanding of the individual's abilities, the support available within their family and other social networks, and the wider resources available within the local community (community assets). The support provider is actively looking to develop creative support plans that make use of the individual's support network and wider community assets, and the individual or their advocates recognise the value of using community resources where possible in the support plan. There is a wide range of accessible venues and activities in the local community and there are frequent opportunities to review the suitability of the support arrangements. *Mechanism offer*: The individual or their advocates have the opportunity to choose activities or support options which involve people from their social networks, and activities within their local communities where appropriate. *Mechanism response*: The individual and their advocates feel confident that the support provider knows them well and the support options they offer are likely to be suitable so they feel confident to try new and innovative community‐based support options. *Outcome*: The individual has the opportunity to get involved with activities within their local community The individual has the opportunity to try new activities and meet new people Community assets see that adults with intellectual disabilities are using their services and make changes so their services are more accessible Community assets become more accessible for a range of different people Using resources that are available to everyone may cost less than bespoke services for adults with intellectual disabilities leading to increased efficiency Using resources that are available to everyone increases the range of options available to adults with intellectual disabilities If adults with intellectual disabilities are supported by people within their social network and local communities they are less reliant on staff within the social care sector If adults with intellectual disabilities are less reliant on staff within the social care sector it reduces the demand across the sector/workforce If support providers can subcontract to people within the individual's support network it means the individual is supported by people that know and care about them and it means those people can be paid for some of the support they provide	Focused search: Animate Consulting 2015, Ridley et al. 2012; Thistle Foundation Animate 2014 Online real‐world stories: Debbie's story: https://citizen‐network.org/uploads/attachment/841/debbies‐individual‐service‐fund‐story.pdf Luke's story: https://www.youtube.com/watch?v=C_BPROKpqXM&t=17s Trudy's story: https://www.youtube.com/watch?v=hnfe6SMmoZk Advisory network contribution: Advisory Group meeting (June, 23); adult with intellectual disabilities/support worker workshop (July, 23); Stakeholder interviews: Academic expert, provider, commissioner, social worker Stakeholders example quote: There are some people who will always need some support and maybe because of somebody's learning disability they will always need some level of paid support but how do we make that as little as possible because you're getting what you need because you've got mates that you can go to the pub with, or you're doing voluntary work and, or you're going to church but you don't need a member of staff to go with you because you're car sharing with the person down the road, who's your neighbour, kind of thing. So it's important because people should be active citizens in their community, and feel part of it and feel about, be valued for kind of who they are and what they're offering. (Care provider manager participant 5, stakeholder interviews)
7 ISF providers take a positive approach towards risk	7.1 Providers supported to be positive about risk *Context*: the Local Authority trusts and sanctions providers to assess the risk of different support options offered to an individual, and to weigh these against the benefits for that individual when considering different support options. The providers have a good understanding of their legal duty of care and the requirements of the Care Act and are looking to provide new opportunities for individuals. The providers have the time and skills to assess risks in specific situations, to communicate the risks and benefits of different activities and support options and they involve the individual or their advocates in decisions about the level of acceptable risk *Mechanism offer*: The individual or their advocates have the opportunity to contribute to decisions about the levels of acceptable risk, with support to understand where, how and if, any risk can be mitigated. *Mechanism response*: The individual or their advocates feel confident to try new activities, experiences or support options because they understand the risk involved and they trust the provider to take action to reduce the risk where possible and to revise the support plan quickly if they change their mind about the option or it does not work well. *Outcomes*: The individual is supported to try new experiences, activities and support options leading to a more interesting and varied support plan The individual has the flexibility to try new things because risk is no longer a barrier The support options may promote independence and be less restrictive The providers can be more creative in the way they meet support needs and address the required outcomes The individual may be more involved with their local community and able to broaden their social network	Focused search: Animate Consulting 2015; Thistle Foundation Animate 2014 Online real‐world stories: Callum and Moira's story: https://vimeo.com/162070462 Advisory network contribution: Advisory Group meetings (June, 23; July, 23; January, 24) Stakeholder interviews: Provider, family carer Stakeholders example quote: Yeah, you can do that. Yeah, it's dangerous but we will risk assess it. Make sure that we've thought about all the risks. If staff can see that happening in their daily lives, then they're more likely to believe they can do it, but they need to be empowered as well. So if you've got controlling managers and controlling leaders in services, who don't find it easy to trust staff to make some of those decisions themselves, then of course, it's harder for them. So they need space for the right kind of culture to work in where people being confident, take decisions and empowered to take decisions, supported when it goes wrong. And some of that stuff. It's a cultural thing. Isn't it? It's the atmosphere of the service they work in. (Provider ARC Participant 3 stakeholder interviews)
8 Systems that value and nurture trust and positive relationships	8.1 Building and maintaining positive relationships between the provider, the individual and their advocates *Context*: the provider actively seeks to get to know the individual's abilities, preferences and priorities over time, whilst also understanding the proximal and distal outcomes that are important to them and their advocates. Over time the individual and their advocates have positive experiences with the provider listening and responding to their preferences and concerns by creating and continuously seeking to improve a support plan that reflects the decisions they have made about their preferences for support. *Mechanism offer*: The individual and their advocates have regular opportunities to discuss the support plan with someone they know and trust to have the best interests of the individual at heart *Mechanism response*: The individual and/or their advocates feel confident because they know and trust the people who are responsible for holding their budget and planning and overseeing their support. *Outcomes*: The individual and their advocates have a good relationship with the provider that holds their budget and plans the support. The provider continually listens to the individual and their advocates to learn what is important to them Over time there is a virtuous cycle of developing trust and relationship The individual and/or their advocates become more confident to speak up about their preferences and concerns so they have more say in the decisions that are made about support. The provider is able to tailor the opportunities and the support to the individual, based on their increasing knowledge of that individual Reduced anxiety and concern about support for the individual, their family and friends and other advocates More rewarding work for support staff increasing their job satisfaction and the likelihood of them staying in their role supporting the consistency for the individual and their advocates Improved retention of staff and reduced recruitment costs for the provider in the long term	Focused search: Animate Consulting 2015; Ridley et al. 2012; Thistle Foundation Animate 2014 Online real‐world stories: Adam's story: https://citizen‐network.org/uploads/attachment/840/adams‐individual‐service‐fund‐story.pdf Debbie's story: https://citizen‐network.org/uploads/attachment/841/debbies‐individual‐service‐fund‐story.pdf Bev's story: https://citizen‐network.org/uploads/attachment/842/bevs‐individual‐service‐fund‐story.pdf Luke's story: https://www.youtube.com/watch?v=C_BPROKpqXM&t=17s Trudy's story: https://www.youtube.com/watch?v=hnfe6SMmoZk Callum and Moira's story: https://vimeo.com/162070462 Louise's story: https://www.youtube.com/watch?v=Bvqp6d2i_ck Advisory network contribution: Advisory Group meeting (July, 23; September, 23); Adults with an intellectual disabilities/support worker workshops (July, 23; September, 2023; October, 23) Stakeholder interviews: Academic expert, social enterprise founder, providers, commissioner, social worker, family carer Stakeholders example quote: Ultimately, if we actually trust people, to know what they need out of services, they can work really well. social worker participant 8 stakeholder interviews.

Abbreviations: AG, Advisory Group; PMG, Project Management Group.

**FIGURE 3:**
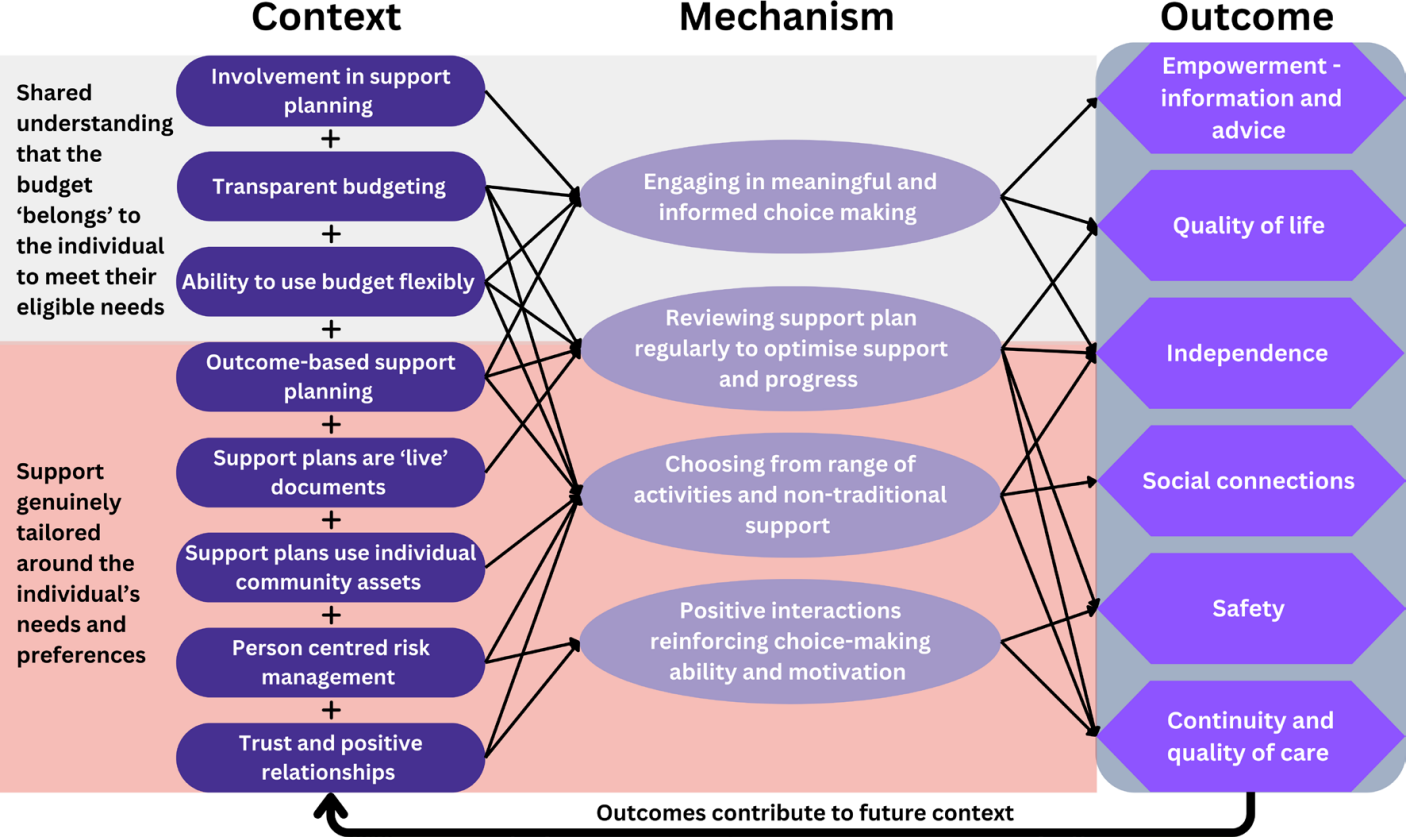
The ISF programme theory.

Our programme theory illustrates how initial programme theories interact within an ISF model to produce positive outcomes for adults with intellectual disabilities and their advocates. These outcomes are aligned with the domains outlined in the ASCOF (ASCOF 2023–2024), which provides a standardised framework for measuring the quality of social care services; see Figure 3.

### 3.2 Initial Programme Theories

#### 3.2.1 Involvement in Support Planning

ISFs work well when social care systems actively involve the individual and their advocates in support planning. This ensures the support plan is built around their abilities, preferences and priorities and addresses outcomes that are important to them. When the ISF provider knows the individual and their advocates well and has the time and the skill to explain potential support options and their implications, and they are also able to provide decision‐making support according to the individual's capacity, then they enable the individual or their advocates to make and express their choice. In this way, ISFs can allow individuals to make a genuine contribution to decisions about their support.

Furthermore, adults with intellectual disabilities and their advocates who have positive experiences with responsive ISF providers begin to realise that they have agency. When providers listen to them and incorporate their preferences and priorities into support plans, individuals understand they can influence the support they receive. This motivates them to engage in the process and offer their opinions. This creates a virtuous cycle of increasing agency over support arrangements. This in turn leads to greater satisfaction and less anxiety about support, which improves quality of life.

#### 3.2.2 Availability of Accessible Information About the Budget

ISFs work well when systems provide accurate, accessible and timely information about the budget. This allows the individual or their advocates to weigh different support options against the budget implications. Having accessible budget information and being supported to use this in decision making allows the individual or their advocates to consider changes to their support arrangements. This gives them the confidence and the ability to make or contribute to decisions because they understand the financial implications of their choices. This allows them to feel in control of the budget and their support arrangements and helps them develop the skills needed to manage a budget.

Receiving clear information from the provider also reassures individuals or their advocates that they are not being taken advantage of. This builds trust between the provider and the individual or their advocates.

#### 3.2.3 Ability to Use the Budget Flexibly

ISFs work well when systems are established to enable ISF providers to offer innovative and creative support options by proficiently managing budgets. This includes using joint purchasing, saving, or sharing budgets, thereby encouraging ISF providers to continuously explore new ways to support people. Where ISF providers know the individual well, they can tailor support options, activities or experiences to that person. They can also provide appropriate support to choose something different, with reassurance that the person can change their mind if the new arrangement does not work well. Additionally, they can give a clear explanation of how various support options could be delivered within the budget. This gives the individual and their advocates the confidence to try something new.

#### 3.2.4 Support Planning Based on Outcomes Rather Than Time and Task

ISFs work well when systems support and promote ISF providers who develop flexible support plans that focus on the outcomes that are important to the individual, rather than on time and task allocations of support. Providers who break down an individual's outcomes into achievable steps and find creative approaches to reach them offer individuals meaningful choices in activities and support options. This outcome‐focused approach enables progress that time‐and‐task‐based support delivery is unlikely to match.

#### 3.2.5 Support Plans as Live Documents That Are Reviewed Regularly and Changed Easily

ISFs work well when ISF providers regularly reassess progress towards outcomes, using both formal and informal methods and are able to make modifications to support plans without the need for a formal review by local authority staff. This enables ‘live’ support plans, which can be adapted quickly in response to changes in circumstances or preferences. This avoids needs escalating as a result of a lengthy wait for formal reassessment by overstretched local authority staff.

#### 3.2.6 Support Plans Make Use of Support Networks and Community Resources

ISFs work well when ISF providers formulate support plans that leverage the individual's support network and wider community assets. This approach allows individuals to try new activities and use non‐traditional support options where suitable opportunities exist in their communities. Support options available in the community may cost less than bespoke services for adults with intellectual disabilities, leading to increased efficiencies and allowing people to make their budget go further. Moreover, if support providers can subcontract to people within the individual's support network, it means that the individual is supported by people who know and care about them, and those people can be paid for some of the support they provide. This in turn reduces the demand on the formal social care workforce.

#### 3.2.7 ISF Providers Take a Positive Approach Towards Risk

ISFs work well when they are delivered within social care systems that endorse positive risk management. These systems need clear lines of responsibility and accountability, with overall risk shared between stakeholders. Each stakeholder should be capable and trusted to make defensible decisions about risks. Systems that support ISF providers in assessing the specific risks and benefits of different support options, and in communicating these, enable the individual or their advocates to contribute to decisions about acceptable risk levels. This gives the individual and their advocates genuine choice over a wider range of opportunities. This genuine choice arises from their understanding of potential risks and their trust in the provider's plans to minimise or mitigate these risks. It also depends on trust that support plans can be revised quickly if they change their minds or if plans do not work well.

#### 3.2.8 Systems That Value and Nurture Trust and Positive Relationships

ISFs work well when providers create regular opportunities to discuss the individual's support plan, their abilities and preferences, and progress towards outcomes that are important to them. When providers use this to continuously improve their support, trust is built because individuals and their advocates see their preferences enacted and support improving. As trust builds, the individual or their advocates gain the confidence to speak up about their preferences and concerns, allowing the provider to better tailor opportunities and support to the individual, based on their increasing knowledge of that person. As support is tailored to the individual's preferences and priorities, quality of life improves. This cyclical process is premised on the idea that the outcomes of one interaction inform or transform the context for subsequent stages. As a result, a virtuous cycle is created, fostering deeper positive relationships, building trust and confidence between the individual, their advocates and the support provider. This, in turn, leads to greater involvement in decision‐making, improved support and better quality of life.

## 4 Discussion

Before discussing the findings of this realist review, it is important to acknowledge the limitations and consider the way we tried to mitigate these. Many included studies were derived from organisations keen to promote the use of ISFs, and the case studies were similarly more likely to highlight positive outcomes. This is not uncommon when using non‐traditional sources, as those invested in the success of an endeavour may be more likely to document and share their experiences; however, it may have resulted in a positive reporting bias. To mitigate this and gain a more balanced perspective, we intentionally included a significant number of stakeholders with ‘real‐world’ experience in developing and delivering ISFs. Many of these stakeholders shared negative as well as positive experiences with ISFs. We paid particular attention to the circumstances that led to negative experiences and used these to build explanations about what could or should have happened differently to achieve a positive outcome (Pawson and Wong 2013). This approach aligns with realist thinking, which posits that a specific context, when present, triggers a causal mechanism that generates an outcome (Pawson et al. 2004), an outcome that would not occur if that context were absent. In this way, we were able to generate ideas about how to realise the potential benefits of ISFs even in difficult circumstances, thereby contributing to a more comprehensive and nuanced understanding of the causal pathways crucial to their success.

A strength of this work is that it presents the first programme theory specifically for ISFs. However, many of our findings converge with those of Fleming et al.'s (2021) realist review of individualised funding, which included, but was not limited to, managed personal budgets in arrangements similar to ISFs.

Both the current study and the study conducted by Fleming et al. (2021) identified that having control over life choices was a key mechanism leading to improved confidence and self‐esteem, with the ability to use budgets flexibly increasing opportunities for social and recreational activities, which in turn enabled people to contribute to and participate in their communities. Similarly, both studies found that a crucial contextual factor enabling this was the availability of a support network. Fleming et al.'s analysis indicated that paid coordinators—which our findings suggest could include ISF providers—contributed to this support network by enabling greater community integration through sourcing information, providing support to try new experiences and meet different people and managing administrative and management tasks.

Furthermore, Fleming et al. (2021) found that collaborative relationships were an important mechanism leading to positive outcomes of shared understanding and continuity of care. Both the current study and Fleming et al.'s study (2021) also highlighted the importance of outcome‐focused support planning and positive approaches to risk management.

Our participatory realist review of ISFs extends Fleming et al.'s work in several key ways: first, by drawing on a diverse range of sources rather than relying solely on published academic literature; second, through its specific focus on ISFs rather than other forms of individualised funding such as direct payments; and third, by comprehensively including adults with intellectual disabilities and other key stakeholders through our advisory network and qualitative research in phase 3.

Our ISF programme theory demonstrates how contexts in which ISF providers have adequate staffing levels and a workforce who know the individual well, as well as the time, skills, knowledge and resources to engage them and/or their advocates in deciding how support will be provided, generate positive outcomes for the individual.

However, whilst the individual's active participation and decision‐making power form the essence of self‐directed support (DeCarlo et al. 2019; Lockman Turner et al. 2022; Lakhani et al. 2016), it is clear to see that the context that enables this is not the context for many support providers, who are operating in a wider context of reduced funding, staffing shortages and increased demand for services (Care Quality Commission 2023). The strain on resources and the need to prioritise basic needs over individualised support may mean that many providers are unable or unwilling to fully engage with individuals and their advocates in the support planning process (Davies et al. 2015; Robertson et al. 2007). Consequently, the context necessary for generating positive outcomes is often compromised, resulting in a gap between the expected and actual quality of care delivered.

Individualised information about the budget and its use, provided in an accessible format, is essential to empower individuals and their advocates to make meaningful decisions about their support. However, this transparency and involvement in decision‐making are contingent on contexts in which all parties understand that the budget belongs to the individual, rather than to the local authority or the provider. This wider context may represent a significant cultural shift in the way social care systems, services and staff conceptualise and manage personal budgets (Humphries 2022; Eriksson 2014).

ISF programme theory highlights that the success of ISF hinges on treating support plans as dynamic and changing ‘live’ documents that require regular review and a creative and flexible approach that consistently seeks to optimise support provision. The ability to change suboptimal care arrangements is crucial to improve the quality of support in the context of a ‘market of care’ (Dursin et al. 2021), underscoring the need for robust mechanisms to support individuals to recognise and respond to poor quality care. Moreover, in the current context, budgets are usually allocated using points‐based resource allocation systems (Series and Clements 2013) which may contribute to ingrained ways of thinking that support must be provided according to specific parameters of time and task, rather than encouraging ISF providers to innovate and use budgets more flexibly than these allocations suggest. This reinforces the importance of agile and responsive channels of communication, shared expectations and trusting relationships in creating contexts in which adults with intellectual disabilities can benefit from the flexibility offered by ISFs. Furthermore, creative and progressive support planning is contingent on sufficient budget allocation to allow flexibility, and adults with intellectual disabilities and their advocates having confidence that the goal is improved support quality and experience rather than budget reduction. This presents something of a challenge in light of current widespread, and well‐founded suspicion of local authorities' cost‐cutting priorities (Beresford and Slasberg 2023).

ISFs provide individuals with the opportunity to choose non‐traditional support options and make use of community assets, resulting in more varied and personalised support plans that foster progress. However, this depends on the availability of a diverse range of accessible resources in the local area (Hummell et al. 2023; Needham 2013), and providers' awareness of these options. This aligns with Fleming et al.'s (2021) realist analysis of individualised funding, which found that access to such funding improved social and recreational opportunities, enhancing self‐image. Key contextual factors for enabling community integration include the ability to purchase services from mainstream, community‐based settings, and the support network around the individual, comprising both unpaid (family and friends) and paid support, including ISF providers.

ISFs enable individuals to try new experiences and activities in contexts where support providers have the skills and confidence to assess risks, implement mitigation strategies and clearly communicate these risks to individuals and their advocates, allowing for informed decision‐making. Fleming et al. (2021) support this finding, indicating that a transparent, positive approach to risk‐taking is essential for successful individualised funding.

ISFs are successful in contexts that value and nurture trust, positive relationships and shared values, alongside a commitment to collaboration between all parties. This context is sustained by social care systems and processes that promote frequent, open communication, align expectations and foster a culture of collaborative problem‐solving and continuous improvement. Realist thinking suggests that the outcomes of initial interactions form the context for future engagements, creating a ripple effect (Jagosh et al. 2015). As trust and collaboration increase, they create a more favourable environment for innovative and effective support. Conversely, deteriorating relationships can lead to misunderstandings or conflicts, impeding progress. This dynamic highlights the importance of nurturing strong relationships and communication over time, as each interaction shapes the context for future collaboration and outcomes within the ISF framework.

### 4.1 Implications and Conclusion

This participatory realist review has produced a programme theory which explains what needs to be in place in order for ISFs to be successful for adults with intellectual disabilities. This theory can be used to guide policymakers, practitioners and support providers to develop, adapt and implement ISFs that have genuine potential to improve support outcomes for adults with intellectual disabilities, enable more efficient resource allocation, reduce workload on overburdened local authority staff and extend the social care workforce. ISFs have the potential to extend the benefits of self‐directed support to people with intellectual disabilities, who might otherwise be confined to a narrow range of segregated, disability‐specific services through council‐managed budgets. ISFs therefore have the potential to reduce inequalities in access to self‐directed support whilst helping people become involved in their communities. Our programme theory elucidates the causal pathways by which ISFs achieve these outcomes for adults with intellectual disabilities. The explicit connection of causal mechanisms to the contextual factors that enable these, allows those offering or delivering ISFs to work towards the conditions essential for the success of ISFs. Furthermore, these findings equip adults with intellectual disabilities and their advocates to judge whether their specific ISF offer provides the context for achieving successful outcomes, helping them to make informed decisions about whether to choose an ISF.

## Author Contributions

E.C. funding acquisition, conceptualised and designed review, carried out focus groups, screened and synthesised data, drafted paper. A.D. contributed to the conception and design of the review, carried out interviews and focus groups, screened and synthesised data, drafted the paper. A.B. funding acquisition, conceptualised and designed the review, ran searches, advised on screening and synthesis, commented on drafts. C.T. funding acquisition, produced accessible research materials, contributed to focus groups and data synthesis, drafted paper.

## Funding

This research was funded by the National Institute for Health Research [Health and Care Delivery Research NIHR151776]. The views expressed are those of the authors and not necessarily those of the NIHR or the Department of Health and Social Care.

## Ethics Statement

Ethics approval was granted by the University of Sheffield, School of Health and Related Research ethics committee (20/07/23; REF: 054823).

## Conflicts of Interest

The authors declare no conflicts of interest.

## Data Availability

The qualitative data set is available from the corresponding author upon reasonable request.
